# Retrospective investigation of combination therapy with clarithromycin and levofloxacin for pulmonary *Mycobacterium avium* complex disease

**DOI:** 10.1186/s40780-015-0025-4

**Published:** 2015-09-03

**Authors:** Hitoshi Shimomura, Airi Ono, Keiko Imanaka, Toru Majima, Hidenori Masuyama, Tsugumichi Sato, Takao Aoyama

**Affiliations:** Faculty of Pharmaceutical Sciences, Tokyo University of Science, 2641 Yamazaki, Noda, Chiba 278-8510 Japan; Department of Pharmacy, Chemotherapy Research Institute, Kaken Hospital, 6-1–14 Konodai, Ichikawa, Chiba 272-0827 Japan; Department of Respiratory medicine, Chemotherapy Research Institute, Kaken Hospital, 6-1–14 Konodai, Ichikawa, Chiba 272-0827 Japan

**Keywords:** *Mycobacterium avium* complex, Levofloxacin, Clarithromycin, Clinical efficacy, Bacilli negative conversion rate

## Abstract

**Background:**

Fluoroquinolones are often used for the treatment of refractory *Mycobacterium avium* complex (MAC) disease when the clinical efficacy of the recommended regimen, which includes clarithromycin (CAM), rifampicin (RFP), and ethambutol (EB), is insufficient. However, recent *in vitro* and *in vivo* studies have suggested that fluoroquinolones decreased the antibacterial activity of CAM when they were administered in combination. In this study, we retrospectively investigated the influence of the combination of CAM and levofloxacin (LVFX) on clinical outcomes for pulmonary MAC disease patients.

**Methods:**

Pulmonary MAC disease patients from 2010 to 2012 were divided into two groups, those who received LVFX together with CAM (LVFX group) and those who received CAM without LVFX (control group). The number of patients who showed improvement was evaluated at 1, 3, 6 and 12 months after the start of therapy based on bacteriological examination (culture and smear examination) and the bacilli negative conversion rate.

**Results:**

There were no significant differences between the LVFX group (*n* = 18, 64.5 ± 6.5 years old) and the control group (*n* = 57, 71.0 ± 7.0 years old) in terms of gender, age, etiologic agent, baseline culture examination score, concomitant medication, and dosage of each drug. The clinical outcomes in the LVFX group were inferior to those in the control group at all endpoints and observational periods, and we found a significant difference in the percent improvement of the smear examination by fluorescence microscopy method (38 % vs. 83 %) and the bacilli negative conversion rate (38 % vs. 79 %) at 3 months. Our study suggests that the combination of CAM and LVFX causes unfavorable clinical outcomes for pulmonary MAC disease treatment. There was no significant difference between both groups in terms of frequency of adverse events.

**Conclusion:**

The possibility that combined administration of CAM and LVFX causes unfavorable clinical outcomes for pulmonary MAC disease treatment was suggested.

## Background

*Mycobacterium avium* complex (MAC) is the most common etiologic agent in lung disease caused by nontuberculous mycobacteria [1]. Because MAC is not susceptible to antituberculous drugs, clarithromycin (CAM) is the key drug for treatment of pulmonary MAC disease, and multidrug therapy with rifampicin (RFP) and ethambutol (EB) is recommended [2, 3]. CAM inhibits protein synthesis by preventing the activity of the 50S ribosomal subunits of bacterial 70S ribosomes. RFP and EB are used together to prevent the formation of drug–resistant bacteria. However, the bacilli negative conversion rates of this therapy are reported to be approximately 60–80 % [4–8]. Therefore, this disease is often difficult to treat. In difficult–to–treat cases or when the recommended drugs cause side effects, fluoroquinolones (FQ) are frequently used. FQ inhibit bacterial DNA gyrase or the topoisomerase IV enzyme, thereby inhibiting DNA replication and transcription. Since FQ have been demonstrated to have antibacterial activity against MAC both *in vitro* and *in vivo*, it could be expected to demonstrate high efficacy for the treatment of pulmonary MAC disease [9, 10].

Several studies have reported no differences in outcomes between the FQ–containing regimen and the CAM, RFP, and EB combination therapy [11–13]. However, recent *in vitro* and *in vivo* studies have suggested that fluoroquinolones decreased the antibacterial activity of CAM when they were administered in combination [14]. Therefore, this drug combination may cause unfavorable clinical outcomes. Currently, few clinical reports have examined the curative effects of CAM and FQ combination therapy for MAC disease. Moreover, only extremely limited data are available regarding levofloxacin (LVFX) in particular. In 2005, Taga and colleagues reported that clinical outcomes did not improve even if LVFX was added to the three–drug treatment of CAM, RFP, and EB [13]. In Japan, health insurers approved the use of 500 mg/day of LVFX in 2009 and 800 mg/day of CAM in 2008; therefore, a higher dose regimen is used for each drug than before. Consequently, treatment outcomes in earlier studies based on the previously recommended dose may be different from those based on the current recommended dose.

In this study, we retrospectively investigated the clinical outcomes of pulmonary MAC disease patients administered the current recommended dose of CAM and LVFX from 2010 to 2012, and examined the therapeutic efficacy of the combination of CAM and LVFX.

## Methods

### Subjects

From 2010 to 2012, 75 outpatients who were diagnosed with pulmonary MAC disease and received CAM at the Chemotherapy Research Institute, Kaken Hospital (Kaken hospital) were examined in this study. All patients were required to fulfill Japanese Society for Tuberculosis and Japanese Respiratory Society criteria. The patients were classified into two groups; those that received LVFX together with CAM (LVFX group) and those that received CAM without LVFX (control group). Patients with no bacteriological examination results in their medical charts, treated for less than one month, or who were culture negative at the start of therapy were excluded. For patients where LVFX was withdrawn, the subsequent data were excluded.

This study was conducted in compliance with the ethical guidelines for clinical studies and it was approved by the Ethical Review Committee on Clinical Research at Tokyo University of Science (approval and study number: 12006) and Kaken Hospital (approval and study number: 12), and registered in University hospital Medical Information Network–Clinical Trial Registry (April 24, 2013, ID: UMIN000010588).

### Rates of improved patients in the bacteriological examination

Clinical improvement was evaluated by bacteriological examination (culture and smear examination) at 1, 3, 6, and 12 months from the start of therapy. If the score of each examination improved from the last time or remained negative, this was classed as “improvement.” Each percent improvement was calculated by dividing the number of improved patients by the total patients at each time point. The score of each examination was recorded as follows: culture examination, 0 (negative), 1+, 2+, 3+, 4+; Ziehl–Neelsen staining method, Gaffky number 0 (negative) –10; fluorescence microscopy method, 0 (negative), 1+, 2+, 3 + .

### Bacilli negative conversion rates in the bacteriological examination

Bacilli negative conversion was evaluated at 1, 3, 6, and 12 months from the start of therapy. If the results of both the culture and smear (Ziehl–Neelsen staining and fluorescence microscopy method) examinations were negative, this was classed as “Bacilli negative conversion.” The bacilli negative conversion rate was calculated by dividing the number of bacilli negative converted patients by the total number of patients at each point.

### Statistical analysis

The baseline patient characteristics data are shown as median ± quartile deviation in age, and mean ± standard deviation in culture examination score. Comparisons of baseline patient characteristics by age and culture examination score were performed using the Mann–Whitney U test. Comparisons of baseline patient characteristics by gender, etiologic agent, concomitant medication, dosage of each drug, percent improvement, bacilli negative conversion rate in the bacteriological examination, and frequency of adverse events were performed using Fisher’s exact test. A P–value of less than 0.05 was considered statistically significant.

## Results

### Patient characteristics and dosage

A total of 75 patients were included in this study, classified as those that used LVFX together with CAM (LVFX group; 18 patients) and the non–combination group (control group; 57 patients), and the background of both groups was compared (Table 1). There was no significant difference between both groups in gender, age, etiologic agent, baseline culture examination score, and concomitant medication. The daily doses were CAM 400–800 mg, RFP 300 or 450 mg, EB 500–1000 mg and LVFX (LVFX group) 300–500 mg based on the opinions recommended by Japanese Society for Tuberculosis and Japanese Respiratory Society (CAM; 15–20 mg/kg, RFP; 10 mg/kg, and EB; 15 mg/kg). There were no significant differences between both groups in dosage of each drug (Table 2).

**Table 1:** Comparison of baseline characteristics of patients with pulmonary *Mycobacterium* *avium* complex disease between the control and levofloxacin (LVFX) groups

Characteristics	Control group (*n* = 57)	VFX group (*n* = 18)
Gender (male/female)	15/42	1/17
Age (years)	71.0 ± 7.0	64.5 ± 6.5
*M.avium/M.intracellulare*	51/9	16/2
Culture examination score	1.04 ± 0.38	1.06 ± 0.64
Other medication	35 (61)	11 (61)
Digestive organ agents	30 (53)	5 (28)
Respiratory organ agents	15 (26)	6 (33)
Vitamins	4 ( 7 )	2 (11)
Allergic agents	4 ( 7 )	1 ( 1 )
Cardiovascular agents	4 ( 7 )	0 ( 0 )
Anti-inflammatory agents	3 ( 5 )	2 (11)
Hypnotics and sedatives, anxiolytics	3 ( 5 )	0 ( 0 )
Miscellaneous	2 ( 4 )	0 ( 0 )

The results are shown as median ± quartile deviation in age, and mean ± standard deviation in culture examination score. Values in other medication express as number (%) of patients. There was no significant difference for each characteristic between both groups

**Table 2:** Comparison of daily doses of clarithromycin (CAM), rifampicin (RFP), ethambutol (EB), and levofloxacin (LVFX) for pulmonary *Mycobacterium avium* complex patients between the control and the LVFX group

Drug	Daily dose (mg)	Control group (*n* = 57)	LVFX group (*n* = 18)
CAM	400	1	1
	600	1	2
	800	55	15
RFP	300	11	2
	450	46	16
EB	500	29	6
	750	26	7
	1000	2	2
LVFX	300	-	7
	375	-	2
	500	-	9

Values express as number of patients. There was no significant difference for each dosage between both groups

### Rates of improved patients in the bacteriological examination for every period

Improvement of culture examination at one month was observed in 50 % of patients in the LVFX group and in 58 % of patients in the control group. The percent improvements at 1 and 3 months in the smear examination (Ziehl–Neelsen staining method) were 61 and 63 % in the LVFX group, and 77 and 90 % in the control group, respectively. The percentage improvement of the LVFX group was always lower than that in the control group at the same time point. Through the whole period, the LVFX group showed a lower percent improvement in the smear examination (fluorescence microscopy method) than that in the control group, and a significant difference was detected at 3 months (38 % vs. 83 %, *P* = 0 .013). Although patient dropout owing to adverse effects or completion of treatment increased with time (approximately half of the LVFX group by 3 months), the percent improvement in each examination of the cases that could be observed until 12 months was more than 65 % (Table 3).

**Table 3:** Comparison of rates of improvement in patients with pulmonary *Mycobacterium avium* complex disease between the control and levofloxacin (LVFX) groups

Evaluation	Group	Follow-up period (months)			
		1	3	6	12
Culture examination	Control	33/57 (58 %)	43/52 (83 %)	38/48 (79 %)	35/44 (80 %)
	LVFX	9/18 (50 %)	4/8 (50 %)	4/6 (67 %)	2/3 (67 %)
Smear examination					
Ziehl–Neelsen’s staining	Control	44/57 (77 %)	47/52 (90 %)	44/48 (92 %)	36/43 (84 %)
	LVFX	11/18 (61 %)	5/8 (63 %)	5/6 (83 %)	2/3 (67 %)
Fluorescence microscopy	Control	41/57 (72 %)	43/52 (83 %)	44/48 (92 %)	38/44 (86 %)
	LVFX	10/18 (56 %)	^*^3/8 (38 %)	4/6 (67 %)	2/3 (67 %)

^*^ A P-value of less than 0.05 was considered statistically significant

### Bacilli negative conversion rates in the bacteriological examination for every period

Bacilli negative conversion rates at 1 and 3 months were 28 and 38 % in the LVFX group, and 49 and 79 % in the control group, respectively. In all periods, the LVFX group exhibited a lower bacilli negative conversion rate than the control group, and a significant difference was detected at 3 months (*P* = 0.026). The bacilli negative conversion rates of the control group and the LVFX group at 12 months were 76 and 67 %, respectively (Table 4).

**Table 4:** Comparison of bacilli negative conversion rates of pulmonary *Mycobacterium avium* complex patients between the control and levofloxacin (LVFX) groups

Group	Follow-up period (months)				
	Start of treatment	1	3	6	12
Control	0/57 (0 %)	28/57 (49 %)	41/52 (79 %)	39/48 (81 %)	32/42 (76 %)
LVFX	0/18 (0 %)	5/18 (28 %)	*3/8 (38 %)	4/6 (67 %)	2/3 (67 %)

If the results of both the smear and the culture examination were negative, the patients were classed as “bacilli negative conversion.” *A P-value of less than 0.05 was considered statistically significant

### Frequency of adverse events

Adverse events were observed in 37 % of patients in the control group and 56 % of patients in the LVFX group. Skin manifestation was the most common adverse events in both groups (control group; 16 %, LVFX group; 22 %). The other adverse events were as follows: visual impairment (12 %), gastrointestinal symptom (11 %), hepatic dysfunction (2 %), and miscellaneous (5 %) in the control group, and gastrointestinal symptom (17 %), visual impairment (11 %), hepatic dysfunction (11 %), and miscellaneous (1 %) in the LVFX group. Each and overall adverse event frequency did not have significant differences between both groups (Table 5).

**Table 5:** Comparison of adverse effect that occurred in patients with pulmonary *Mycobacterium avium* complex disease between the control and levofloxacin (LVFX) groups

Adverse effects	Control group (*n* = 57)	LVFX group (*n* = 18)
Skin manifestation	9 (16)	4 (22)
Visual impairment	7 (12)	2 (11)
Gastrointestinal symptom	6 (11)	3 (17)
Hepatic dysfunction	1 ( 2 )	2 (11)
Miscellaneous	3 ( 5 )	1 ( 1 )
Total	21 (37)	10 (56)

Values express as number (%) of patients. There was no significant difference in total and each adverse effect between both groups

## Discussion

Clinical outcomes of FQ–containing regimens for pulmonary MAC disease have recently been reported worldwide [11–13, 15]. Jenkins and colleagues claimed that there was no difference in outcomes between the RFP + EB + ciprofloxacin and RFP + EB + CAM groups [11]. Fujita and colleagues insisted that the RFP + EB + gatifloxacin regimen demonstrated good efficacy in comparison to the recommend CAM–containing triple–drug combination therapy [12]. Koh and colleagues reported that the addition of moxifloxacin could improve the outcomes in approximately one–third of patients with persistently culture–positive MAC disease who fail to respond to CAM–containing regimens [15]. Concerning LVFX, which is frequently used in Japan, Taga and colleagues reported that clinical outcomes did not improve even if LVFX was added to the three–drug combination treatment of CAM, RFP, and EB [13]. However, clinical outcomes with the current higher dose have not been examined. This is the first study to investigate the clinical outcomes of pulmonary MAC disease patients administered the current recommended dose of CAM and LVFX, and examine the therapeutic efficacy of the combination of CAM and FQ.

Concerning baseline characteristics of patients, there were no significant differences between the LVFX group and the control group in terms of gender, age, etiologic agent, culture examination score, dosage of each drug, and concomitant medication (Tables 1 and 2). In this study, the mean of culture examination score in both groups at the start of treatment were same level. As for the degree of severity in patients of both groups, almost all patients were mild case (culture examination score: 1+). Patients with moderate grade (culture examination score: 2+) were several, and this study did not include severe cases (culture examination score: 3+). Generally, performance status is very important to evaluate the outcomes of chemotherapy for patients with infectious diseases. However, it was less likely to affect the outcomes of this study because all patients were outpatients whose performance statuses were 0 or 1. In addition, patients were started chemotherapy after having checked the laboratory findings of renal and hepatic function. Thus, patients with renal and hepatic disease were excluded. Accordingly, patients in our study seemed to be appropriate.

In the present study, the clinical outcomes in the LVFX group were inferior to the control group at all endpoints and observational periods, and we found a significant difference in the percent improvement of the smear examination by fluorescence microscopy and the bacilli negative conversion rate at 3 months. Through their *in vitro* and *in vivo* studies, Kohno and colleagues demonstrated that antagonism between CAM and FQ could occur when they were administered in combination [14]. Similarly, our present investigation suggested the possibility that the treatment outcome worsened when CAM and LVFX were administered together. The bacilli negative conversion rates at 6 and 12 months in the control group in this study (81 and 76 %, respectively) were higher than those reported by Taga and colleagues (70 and 64 %, respectively) [13]. This may have been because the daily dose of CAM in this study (typically 800 mg) was higher than the conventional daily dose (typically 600 mg). Because it was possible to administer CAM 800 mg/day for treatment of MAC after 2008, Kobashi and colleagues compared the clinical effect of combined chemotherapy (CAM 400 or 600 mg, RFP and EB) containing streptomycin from 1998 to 2007 and combined chemotherapy (CAM 800 mg, RFP and EB) containing streptomycin from 2008 to 2010. According to their results, the bacilli negative conversion rate for each treatment was 69 and 84 %, respectively [16]. This percentage was almost consistent with the bacilli negative conversion rate in the control group at 3 months in this study (approximately 80 %).

However, the results of this study are not sufficient to demonstrate that the combination of CAM and LVFX causes unfavorable clinical outcomes in pulmonary MAC disease patients. It is noteworthy that the percentage of all endpoints in the LVFX group were lower than those in the control group, but drop–out cases in the LVFX group increased with time (approximately half at 3 months) because many patients were withdrawn from the LVFX + CAM combination early in the treatment period. Therefore, larger–scale studies that are neither case–limited nor retrospective are needed to assess the influence of LVFX on the therapeutic efficacy of the recommend CAM–containing triple–drug combination therapy.

On the other hand, similar adverse events were observed in both groups. Each and overall adverse event frequency did not have significant differences between both groups (Table 5). Accordingly, these findings may indicate that the combination treatment of CAM and LVFX is not inferior in safety in comparison with standard treatment.

Currently, FQ are still used as therapeutic drugs for refractory MAC patients because an alternative treatment to the recommended therapy of CAM, RFP, and EB is not established. It has been reported that the *in vitro* and *in vivo* antibacterial activity against MAC as well as the antagonism to CAM of sitafloxacin or moxifloxacin were stronger than those of LVFX [10, 14]. Therefore, the clinical outcomes may be more affected when CAM and these FQ are administered together. MAC originally colonizes the airways, and then infects the epithelial cells and macrophages [17], suggesting that the concentrations of antimicrobials in epithelial lining fluid in lungs are an important factor in its suppression. Recently, a transporter which participates in the transportation of macrolides to epithelial lining fluid of lungs has been reported [18]. It has been also reported that FQ might inhibit the same transporter [19]. Since the interaction mediated by the transporter may be the cause of the antagonism, experimental studies to clarify the mechanism of the decrease in the effect of CAM by FQ combined administration are expected in the future. In addition, further clinical studies on LVFX as well as other FQ are needed.

## Conclusion

This report suggests that combined administration of CAM and LVFX causes unfavorable clinical outcomes for pulmonary MAC disease patients.
